# Experiences of Parents of Children with Cancer During Their Child’s Illness: A Qualitative Interview Study in a Spanish Context

**DOI:** 10.3390/pediatric18050120

**Published:** 2026-09-15

**Authors:** Fernando Santamaría-Fraile, Rosa M. Cárdaba-García, Inmaculada Pérez, Verónica Velasco-González, Baldomero de Maya-Sáchez, Carlos Durantez-Fernández, Lucía Pérez-Pérez

**Affiliations:** 1 Valladolid University Clinical Hospital, 47003 Valladolid, Spain; 2 Department of Nursing, University of Valladolid, 47005 Valladolid, Spainveronica.velasco.gonzalez@uva.es (V.V.-G.);; 3 Nursing Care Research Group, University of Valladolid, 47005 Valladolid, Spain; 4 Valladolid West Primary Care Management (SACYL), 47012 Valladolid, Spain

**Keywords:** neoplasms, pediatrics, hospitalized child, parent, parent–child relationships, psychological stress, coping skills, life-changing events, cancer survivors, death

## Abstract

**Background/Objectives**: Childhood cancer profoundly affects families, particularly parents, who assume demanding caregiving roles associated with significant emotional and social burden that remain underexplored qualitatively. To explore the experiences of parents of children with cancer during the disease process: diagnosis, treatment, follow-up, and their child’s progress. **Methods**: An exploratory qualitative interview study was conducted using individual semi-structured interviews with six parents of children with cancer in Spain. Participants were intentionally recruited through a parent association. Interviews were conducted online, audio- and video-recorded with consent, transcribed verbatim, and analyzed using thematic analysis following Braun and Clarke. Sullivan’s interpersonal theory was subsequently used as a conceptual framework to interpret the emerging themes, particularly in relation to interpersonal anxiety, security, emotional regulation, and significant relationships. **Results**: Six parents were included in the analysis, representing heterogeneous clinical trajectories, including active treatment, remission, and bereavement. Five axes, 12 categories, and 52 codes were identified. Parents described uncertainty and emotional disruption around diagnosis, the coexistence of perceived strength and distress during treatment, changes in family and interpersonal relationships, continuing concerns following remission, and profound grief and continuing bonds following the death of a child. These findings were interpreted through Sullivan’s interpersonal framework. **Conclusions**: This exploratory study suggests that parents’ experiences of childhood cancer are shaped by changes in interpersonal relationships, emotional regulation, perceived security, and support networks across different phases of the illness. The findings highlight potential areas for psychosocial support, although they should be interpreted cautiously given the small and heterogeneous sample.

## 1. Introduction

As per the definition of the National Institutes of Health (NIH), childhood cancer is that cancer that develops between birth and age 14 [1]. Although pediatrics usually begins at birth and ends at 14 years of age, only in the case of pediatric oncology do the units follow these patients until 18 years of age, the age of majority in Spain. In Spain, the National Registry of Childhood Tumors estimates about 1000–1100 new diagnoses every year in the country, representing 0.5–1% of all neoplasms [2]. Although mortality has decreased by 50% in the last 40 years, a cure is not always certain and treatments are long, with prolonged follow-ups, and in many cases, sequelae. Globally, 80% of children reach adulthood free of disease, although survival depends on several factors, such as the tumor type, solid or hematological.

Approximately 1000 children are diagnosed with cancer each year in Spain. Assuming two primary parental caregivers per child, this represents approximately 2000 parents directly involved in caring for a child with cancer. However, it is important to consider other family structures, such as single-parent families, and that in some cases, caregivers are not the only ones involved. Nevertheless, given that the most common family structure is that of two parents, this figure provides a general idea of the number of caregivers most directly affected [2].

The literature indeed shows the stress that caregivers are under throughout the disease process, which sometimes begins even before diagnosis and ends, in the best-case scenario, with the child’s cure, and in the worst one, with the child’s death. The unrelenting exposure to stress increases their vulnerability to physical and emotional ailments [3,4]. Some authors indicate that these parents show feelings of guilt for different reasons, mainly because they feel that they are not fully meeting the demands of their sick children [4,5]. The literature does not contain enough works on this subject to determine exactly what the caregivers’ experience is, since studies have almost always been based on the patients, without taking into account the family unit as the element of analysis.

The classic concept of the mother as primary caregiver of the offspring has been modified in recent decades by the incorporation of women into the workforce and the more active participation of men in child-rearing, so that, although there are increasingly more family models, the classic nuclear family has at least two primary caregivers, who are usually the mother and father [6]. Sometimes other family members, especially siblings who live with the patient, become informal caregivers who must also be taken into account [7].

According to Harry Stack Sullivan, individuals cannot be understood in isolation but rather in an interpersonal context. Based on this theoretical framework, for personality to develop healthily, primary and security needs must be met during childhood. Sullivan’s interpersonal theory was selected as the conceptual framework for this exploratory study because it provides a relational perspective for understanding parents’ experiences in the context of childhood cancer. Rather than assuming that parental distress can be understood solely as an individual response to a stressful medical event, this framework draws attention to interpersonal anxiety, the need for security, emotional regulation, and changes in significant relationships. These concepts were used to guide the interview questions and subsequently to interpret the themes emerging from the qualitative analysis. Given the exploratory nature and heterogeneity of the sample, Sullivan’s framework was used as an interpretive lens rather than as a basis for assuming that all parents experience the illness trajectory in the same way [8].

The interpersonal model of adaptation to disease posits that children who develop a more severe post-traumatic stress disorder after cancer are those whose parents found it more difficult caring for them securely, which emphasizes the need to gain an in-depth understanding of the experiences of parents in order to propose interventions that improve their ability to support their children, reducing distress in both the caregivers and children [9,10].

## 2. Materials and Methods

### 2.1. Design

An exploratory qualitative interview study was conducted using individual semi-structured interviews. The study was designed to explore how parents of children with cancer described and attributed meaning to their experiences throughout different phases of the child’s illness.

Although the study was informed by an interest in the participants’ lived experiences, no specific phenomenological methodology was applied. In particular, the study did not follow the methodological procedures of Interpretative Phenomenological Analysis (IPA), hermeneutic phenomenology, or another distinct phenomenological analytical tradition.

Data were analysed using thematic analysis following the approach described by Braun and Clarke. The resulting themes and categories were subsequently interpreted through Sullivan’s interpersonal theory, with particular attention to interpersonal anxiety, the need for security, emotional regulation, and significant interpersonal relationships.

The study was reported in accordance with the Consolidated Criteria for Reporting Qualitative Research (COREQ) [11].

### 2.2. Research Objectives

To explore the experiences of parents of children with cancer during the course of the disease: diagnosis, treatment, follow-up, and outcome of their child.

To identify the main difficulties encountered by parents of children with cancer and the coping strategies that they use to deal with them, influencing their experiences.

### 2.3. Sample, Recruitment, and Obtention of the Sample

Six interviews were conducted with parents of children with cancer who voluntarily agreed to participate in the study, as the sampling method used was intentional. The sample size was in line with the number of children with cancer in a sparsely populated region in central Spain, but despite this, the size did not depend exclusively on this factor: we also incorporated the principle of data saturation.

Each interview was coded with the letter “E” followed by an ‘O’ if the participant was male and an “A” if female. These vowels were followed by a number from 1 to 6 corresponding to the order in which the interview was conducted.

To participate in the study, the following requirements were met: being the parent of a child with cancer being treated at one of two hospitals in the region of central Spain in Valladolid; volunteer to be interviewed, be able to understand and speak fluently in Spanish, have basic computer literacy, in possession of an electronic device and Internet connection, and agree to be recorded (image and audio) via the Teams^®^ platform. The exclusion criterion was: parents of children with cancer whose mental health was serious.

Six interviews were included in the final analysis. Given the small number of participants and the heterogeneity of their clinical trajectories, the purpose of the sampling process was not to achieve exhaustive saturation across all possible stages or experiences of childhood cancer. Instead, interviews were conducted iteratively, and the research team monitored the development and stability of codes and categories throughout the analytic process.

After each interview, the researchers independently reviewed the transcript and compared emerging codes and categories with those identified in previous interviews. A matrix was used to document the emergence and development of codes, categories, and broader thematic dimensions. Following the fourth interview, no substantially new thematic dimensions were identified, although two additional interviews were conducted to examine the stability and consistency of the emerging thematic structure.

These additional interviews did not result in the identification of substantially new categories. However, given the small and heterogeneous sample, this finding is interpreted as an indication of thematic stability within the experiences represented in the sample rather than as evidence of comprehensive thematic stability across the childhood cancer trajectory. In particular, the study does not claim subgroup saturation for parents experiencing active treatment, remission, or bereavement.

The sample was approached through contact with the Campanilla Association of Children with Cancer. The organization contacted parents who met the inclusion criteria by telephone. Once they agreed to participate, they received information about the study and the informed consent form so that the interview could be conducted on a date and time chosen by the participant. The semi-structured interviews were carried out from February to April 2025.

### 2.4. Data Collection

Individual semi-structured interviews were conducted online using the Teams^®^ platform, with the participants’ prior consent to record image and audio. These interviews lasted 30–70 min. The semi-structured interview guide was informed by Sullivan’s interpersonal framework, particularly the concepts of interpersonal relationships, anxiety, security, and emotional regulation. Questions therefore explored the participants’ experiences and changes in their relationships with their child, partner, family members, healthcare professionals, and other significant people, as well as their experiences of emotional expression, perceived support, and interpersonal changes throughout the illness trajectory. Before the study itself, we undertook two pilot interviews with volunteers from the Campanilla association, which we then used to refine the interview outline. The interviews were carried out exclusively by two members of the research team with previous personal experience of childhood cancer to avoid variability in data collection. Only the interviewer and interviewee were present during the interviews. Field notes were taken at that time, which later allowed us to clarify certain issues that could have been a source of questions or discrepancies. In all cases, the interview started with the collection of sociodemographic and clinical data, followed by questions about the experience researched.

The researchers who conducted the interviews had personal experience with childhood cancer, which was an important element in shaping their positioning in relation to the phenomenon under investigation. This experience potentially facilitated empathy, sensitivity to the participants’ emotional experiences, and the establishment of a trusting environment during the interview. However, it could also have influenced the researchers’ expectations, questions, emotional responses, and interpretation of the participants narratives. Rather than assuming that these influences could be eliminated entirely, the research team adopted a reflective approach during data collection and analysis. The interviewers were aware of their assumptions and personal experiences and reflected on how these might influence their interaction with the participants and their interpretation of their accounts. Regular debriefing sessions were held after the interviews in which the interviewers discussed their emotional responses, assumptions, potentially leading questions, and emerging interpretations. Special attention was paid to distinguishing the participants’ meanings from interpretations potentially influenced by the researchers’ personal experiences. Furthermore, the emerging analysis was discussed with co-authors who had not conducted the interviews. This reflective process was used to enhance the credibility, verifiability, and transparency of the findings.

### 2.5. Data Analysis

The recordings of the interviews conducted in Teams^®^ were stored encrypted in One Drive^®^. All were transcribed verbatim and incorporated into Atlas. Ti^®^ v25 for data management. Two authors were responsible for coding the interviews independently, settling any discrepancies on the basis of the interviewers’ field notes and by a third researcher. Thematic analysis was carried out following the method described by Braun and Clarke, beginning with a reading of the material, generating 52 initial codes that expressed the evoked meaning, and organizing them into 12 thematic groups or categories that generated 5 axes. The content was then organized into categories and the material was analyzed as a whole.

Sullivan’s interpersonal theory was adopted as the theoretical framework underpinning the interpretation of the participants’ narratives. Particular attention was paid to interpersonal anxiety, the need for security, significant interpersonal relationships, emotional regulation, and changes in the interpersonal environment throughout the child’s illness trajectory. These theoretical concepts informed the interpretation of emerging categories while allowing the participants’ accounts to remain grounded in their lived experiences.

### 2.6. Participant Validation

Participant validation (member checking) was conducted after transcription of the interviews. The transcripts were returned to the respective participants so that they could review whether their accounts had been accurately captured and whether the content reflected their experiences, perceptions, and meanings.

Participants were given the opportunity to identify transcription errors, clarify statements, expand on aspects they considered insufficiently represented, or request modifications when they considered that the transcript did not accurately reflect what they had expressed. Any observations received were reviewed by the research team and incorporated into the transcript and/or subsequent analytical process when appropriate.

This procedure provided an additional opportunity to compare the researchers’ preliminary understanding with the participants’ own perspectives and contributed to the credibility and transparency of the analytical process.

### 2.7. Rigor and Trustworthiness

We followed the guidelines of Guba and Lincoln (1985) [12] to ensure the scientific rigor of the study.

The trustworthiness of the study was addressed through several complementary strategies. The study was reported in accordance with the COREQ guidelines [11]. Credibility was supported through investigator triangulation, independent coding by two researchers, discussion of discrepancies with a third researcher, participant validation of transcripts, and reflexive discussions throughout the research process.

Two researchers independently coded the interviews and compared their interpretations. Discrepancies were discussed within the research team and resolved through consensus, with the involvement of a third researcher when necessary. In addition, researchers maintained reflexive awareness of their positioning and personal experiences related to childhood cancer. Regular debriefing sessions were used to discuss assumptions, emotional responses, potentially leading questions, and emerging interpretations. Researchers who had not conducted the interviews also participated in discussions of the emerging analysis.

These procedures were used to enhance the credibility, dependability, confirmability, and transparency of the findings.

### 2.8. Ethical Aspects

This study was approved by the Research Ethics Committee of the Health Catchment Area of Valladolid under code 25-002.

Informed consent was requested from all parents via an informative document provided to the subjects of the study.

Participants were informed of compliance with the ethical principles of confidentiality, personal data protection, and guarantee of digital rights valid in Spain [13]. In addition, the study was conducted in accordance with the principles of Declaration of Helsinki [14] and Regulation 2016/679 of the European Parliament and Council of 27 April 2016 [15], on the protection of natural persons with regard to the processing of personal data and on the free movement of such data. Participation in the study was anonymous and voluntary, and the confidentiality of the information collected was guaranteed.

Only the researchers had access to the personal data and were authorized to process them. The interview data were collected and encrypted on One Drive^®^: a log-in was required to access them.

The recordings were destroyed after the completion of the study, and the participants informed.

## 3. Results

### 3.1. Characteristics of Participants

Out of the total of six participants recruited who met the inclusion criteria, one-third were mothers (33.33%) and two-thirds were fathers (66.66%), according to their official gender. All participants had or had had a partner during the process, as well as other children and family support. The median age of the participants was 39.5 years. The age range was 7 years. The minimum age was 36 years and the maximum was 43 years. The remaining characteristics related to the parents and their children’s illness—given the significant influence they exert on their children and the extent to which their experience of the disease depends on them—are detailed in Table 1. The children were evenly distributed by gender: 50% were boys and 50% were girls. Of these children, two had a hematological malignancy and the remaining four had a solid tumor. Of the six children, one had died, two were still undergoing treatment, and three were in recent remission with regular follow-up.

### 3.2. Axes and Categories Generated

Analysis of the six interviews resulted in five axes, 12 categories, and 52 codes. These categories represent experiences identified within the participants included in the study and should be understood in relation to the heterogeneity of their children’s diagnoses, treatment trajectories, and current clinical status. The findings therefore describe recurrent meanings within the sample rather than exhaustive or universally applicable stages of parental experience. The data are presented in Table 2.

#### 3.2.1. Diagnostic Axis: From Unsettling Ambiguity to Acute Relational Shock

As detailed in Table 2, the diagnostic trajectory unfolded in two primary phases: pre-diagnostic uncertainty and diagnostic confirmation. The narrative across participants reveals that the onset of non-specific symptoms disrupted the baseline relational security of the family long before a formal label was applied. Parents experienced significant interpersonal friction with primary care providers due to initial diagnostic delays, which heightened intuitive anxiety.

Upon diagnostic confirmation, parents suffered an immediate, acute emotional collapse (e.g., EA2, EO1). Rather than acting merely as cognitive avoidance, denial served as a primary Sullivanian interpersonal defense mechanism, allowing parents to temporarily buffer overwhelming threat and remain functionally present for their child during emergency hospital admissions.

#### 3.2.2. Treatment Axis: The Interpersonal Tension Between External Strength and Internal Despair

As detailed in Table 2, the active treatment phase is defined by a fundamental interpersonal duality. Rather than experiencing positive and negative aspects as separate phenomena, parents navigated a continuous tension between projecting external resilience for their child and enduring profound internal distress in isolation.

To maintain relational security and shield their child from existential anxiety, participants described mobilizing emotional strength, accepted the medical reality, and placed deep trust in the clinical staff. This protective buffering manifested through active emotional responses, such as normalizing daily routines for healthy siblings and performing symbolic acts of solidarity—such as a father shaving his own head alongside his son (EO1). Within this protective framework, the inpatient setting itself was reframed from a place of illness into a secure sanctuary that offered peace of mind (EO3).

However, sustaining this external facade of unwavering strength required severe internal sacrifice. To protect their child’s emotional well-being, parents deliberately suppressed their own grief, anger, and feelings of helplessness. Parental distress was forced into private spaces: mothers waited for their children to fall asleep before allowing themselves to cry (EA4), while fathers compartmentalized severe anxiety and repressed raw emotions (EO1, EO5).

This emotional suppression was further exacerbated by the physical reality of treatment toxicities, pervasive fears of child mortality, and acute nocturnal loneliness in the hospital setting (EO1, EO6). Consequently, the treatment phase constituted a delicate balancing act where external strength and internal despair coexisted as interdependent responses mechanisms.

#### 3.2.3. Psychosocial Dimension Axis: Systemic Disruption, Domestic Restructuring, and External Support Networks

As detailed in Table 2, the diagnosis of pediatric cancer triggers an immediate, systemic disruption that reshapes the entire family architecture. Rather than operating as a cohesive household, families are forced into severe structural fragmentation to balance hospital care, employment, and domestic duties.

To maintain daily operations, parents underwent prolonged physical separation: typically, one parent remained full-time in the hospital while the other managed employment and domestic life. This fragmentation extended directly to healthy siblings, whose routines were abruptly altered through long-term relocation to extended family households—such as living with grandparents for months at a time (EO3). Managing this double burden required major professional sacrifices, including formal leaves of absence or quitting jobs entirely (EA2), which, combined with out-of-pocket expenses, resulted in severe financial depletion (EO1). To mitigate this continuous strain, parents relied on brief solitary activities (e.g., running) as vital response mechanisms to temporarily restore emotional equilibrium (EA4).

Crucially, as families navigated these structural and financial strains, significant deficits in hospital-provided mental health services emerged.

Participants described perceived gaps in proactive and integrated psychological support within the healthcare context. Non-governmental parent associations and peer support groups directly filled this institutional void. Beyond logistical and financial relief (EO6), peer interactions offered a unique form of relational validation that the medical system could not supply. Interacting with other parents facing similar life-threatening realities provided mutual empathy and emotional decompression, grounded in the shared understanding that only those living through the crisis could truly comprehend it (EO6).

#### 3.2.4. Remission Axis: The Paradox of Medical Discharge and “Scanxiety”

As detailed in Table 2, the end of active treatment introduces a profound psychological paradox for families. While remission is medically celebrated as a milestone, parents experience it as a transition marked by vulnerability and a sudden loss of institutional protection.

The cessation of active therapy creates an unexpected emotional void. During treatment, the hospital environment and continuous medical surveillance function as a protective sanctuary; once discharged, parents describe feeling abruptly cut off from this safety net, leaving them to navigate recovery without daily clinical oversight.

Rather than bringing complete psychological relief, remission shifts the nature of parental anxiety into a persistent, lingering state. This ongoing threat manifests most acutely around periodic medical check-ups and follow-up imaging—a phenomenon known as “scanxiety”. As scheduled appointments for blood tests or MRIs approach, fear of disease recurrence is reignited (EO1), demonstrating that the emotional toll of childhood cancer persists long after the physical treatment has ended.

#### 3.2.5. Bereavement Axis: The Ultimate Interpersonal Disruption and Spiritual Legacy Transcending Loss

As detailed in Table 2, the death of a child constitutes the ultimate, irreversible disruption of the parental interpersonal sphere. Rather than signaling an absolute termination of the parent–child bond, bereavement forces a profound restructuring of the relationship from a physical reality to a symbolic, spiritual connection.

The immediate aftermath of loss triggers existential despair, with parents expressing an intense desire to join their deceased child (EO5) as a reaction to the sudden collapse of their primary caregiving role. This raw grief is counterbalanced by the final protective act performed during the terminal phase: active participation in end-of-life decision-making. By prioritizing the child’s comfort through the limitation of therapeutic efforts, parents exercise their protective duty until the very end, ensuring a peaceful transition free from unnecessary suffering (EO5).

Following the physical loss, the interpersonal bond is renegotiated rather than severed. Parents maintain an active, ongoing relationship with their child through spiritual presence, internal dialogue, and perceived protective guidance from above (EO5). Crucially, this enduring bond expands outward into a lasting legacy. The child’s impact continues to resonate deeply within the family and broader community, transforming the experience of loss into an enduring memory that preserves the child’s identity and life meaning.

**Figure 1:**
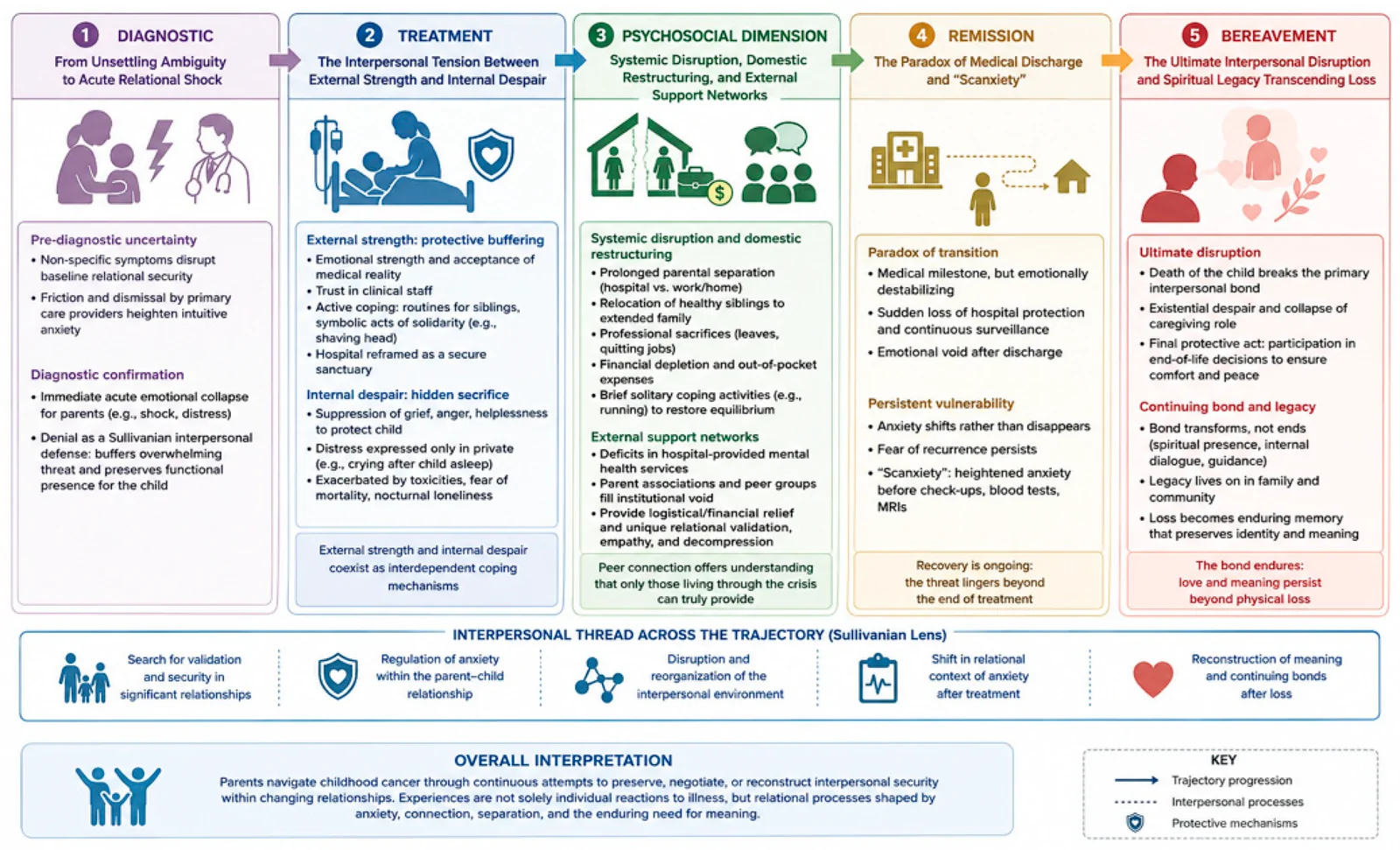
Themes identified in the parents’ accounts across different phases of the childhood cancer experience: an interpersonal interpretation.

The final result of the analysis is a diagram that summarizes all the experiences related to the disease process, as shown in Figure 1.

## 4. Discussion

This study provides a qualitative exploration of the lived experiences of parents navigating childhood cancer. Grounding the analysis in Harry Stack Sullivan’s interpersonal theory offers fresh theoretical insight into how parental anxiety, security, and coping are continually constructed through relational dynamics. Sullivan emphasized that psychological threats disrupt an individual’s interpersonal security system, triggering defense mechanisms and reshaping relational interactions [8]. In pediatric oncology, the child’s diagnosis represents a profound threat to the family’s security baseline, forcing parents to rapidly adapt their interpersonal roles [16,17].

During the pre-diagnostic and diagnostic phases, parents encountered significant relational friction with healthcare gatekeepers [18]. Consistent with previous literature, primary care providers sometimes downplayed or attributed early non-specific symptoms to more common childhood conditions, reflecting the clinical tendency to consider serious illness relatively uncommon in pediatric populations. From an interpersonal perspective, these encounters may be understood as an early disruption of the parents’ interpersonal security. Parents entered the healthcare system seeking reassurance and confirmation that their child was safe; however, when their concerns were not initially validated, uncertainty and anxiety were reinforced through interactions with significant healthcare figures. Thus, the distress experienced during this phase was not solely related to the child’s symptoms, but also to the parents’ perception of whether their concerns were recognized and legitimized within their interpersonal environment [19].

When the diagnosis was finally established, the sudden perception of a threat to the child’s survival generated profound interpersonal shock, and in some cases, denial. From Sullivan’s perspective, anxiety is fundamentally interpersonal and emerges when an individual’s sense of security is threatened within significant relationships [20,21]. Accordingly, parental denial should not be interpreted merely as an individual psychological defense mechanism. Rather, it may be understood as an adaptive interpersonal response that temporarily limits exposure to overwhelming anxiety. By creating psychological distance from the full implications of the diagnosis, denial may protect parents from an abrupt escalation of anxiety and allow them to preserve sufficient emotional functioning to remain available to their child. In this sense, denial can be interpreted as a short-term mechanism for maintaining interpersonal security at a moment when the parent–child relationship is confronted with an existential threat [22,23].

During active treatment, parents navigated a persistent duality between external strength and internal despair. This finding can be particularly well-understood through Sullivan’s emphasis on interpersonal relationships as central to emotional regulation [8]. Parents described suppressing fear, sadness, and crying in the presence of their children, often releasing these emotions only when alone. Such emotional suppression may represent an attempt to protect the child from additional anxiety and preserve a sense of safety within the parent–child relationship [24,25,26]. These accounts may be interpreted as suggesting that parents experienced themselves as both recipients and regulators of interpersonal anxiety: while experiencing intense fear themselves, they attempt to prevent that anxiety from being transmitted to the child. From a Sullivanian perspective, this process illustrates how the need to maintain security within a significant interpersonal relationship may lead individuals to modify their emotional expression, even when doing so entails considerable personal cost [27].

However, this protective interpersonal functioning also had important consequences for the parents themselves. By concealing distress and limiting opportunities for emotional disclosure, parents experienced loneliness, emotional fatigue, and a sense of having to remain strong for others. The findings therefore suggest a tension between interpersonal security and intrapersonal emotional burden. What is protective for the child may simultaneously become psychologically costly for the parent [25,26]. This interpretation may complement previous research by highlighting that parental emotional suppression during pediatric cancer treatment should not be viewed only as an individual’s experiences, but also as a relational process shaped by the parent’s perceived responsibility to preserve the child’s emotional security [28].

Furthermore, the contextual impact of the COVID-19 pandemic must be critically considered when interpreting parents’ experiences during active treatment. Infection control protocols imposed during this period, such as strict isolation within patient rooms, the ban on visitors, and restrictions to a single caregiver without spousal rotation in some cases, acted as a significant contextual amplifier of caregiver stress. These health restrictions significantly exacerbated basic feelings of confinement, loneliness, and emotional exhaustion, temporarily disrupting informal support networks at a time when interpersonal safety was most vulnerable [29].

The gender composition of the sample should also be considered when interpreting the emotional themes identified in this study. Fathers comprised 66.67% of the participants (4/6), while mothers comprised 33.33% (2/6). This contrasts with the predominance of mother-centered samples frequently reported in the pediatric oncology literature and may have contributed to the relevance of themes such as anger, emotional suppression, and the need to project external strength. Fathers may experience additional, socially and culturally shaped expectations to remain strong, protect their child and partner, maintain family functioning, and limit the expression of vulnerability. Such expectations may contribute to emotional suppression or the expression of distress through emotions such as anger. However, these interpretations should be taken with caution, given the small sample size. Rather, the findings highlight the importance of more explicitly including fathers in pediatric oncology research, where their emotional experiences may still be comparatively underrepresented [30,31]. From a Sullivanian perspective, these patterns of emotional expression, potentially marked by gender, can also be understood in relation to interpersonal expectations and the perceived need to maintain security within meaningful relationships.

The gender composition of the sample should also be considered when interpreting the findings. Four participants were fathers and two were mothers. The presence of four fathers provides a perspective that is less frequently represented in some pediatric oncology studies, but the sample is too small to support conclusions about gender-specific differences in coping or emotional expression.

Some accounts in this study involved task-oriented responses, emotional suppression, perceived responsibility for maintaining family functioning, and prolonged caregiving at the bedside. These experiences were described by individual participants and may be shaped by gendered social expectations, parental roles, family circumstances, and the particular phase of the child’s illness. However, the present study cannot determine whether these patterns are systematically associated with being a father or a mother.

Accordingly, the findings should be understood as illustrating diverse parental experiences rather than establishing distinct maternal and paternal coping patterns. Future qualitative studies with larger and more deliberately balanced samples should examine how gender, parental roles, employment arrangements, caregiving responsibilities, and sociocultural expectations interact in shaping parents’ experiences of childhood cancer [8,30,32,33,34,35,36].

Hospitalization further disrupted parents’ interpersonal networks and ordinary patterns of interaction. Spousal separation, relocation of healthy siblings, disruption of employment, and reduced contact with wider family networks altered the interpersonal environment in which parents normally obtained emotional support. From Sullivan’s perspective, these disruptions are particularly relevant because interpersonal relationships constitute a major source of security and emotional regulation. Consequently, the hospital environment may simultaneously provide highly specialized medical support while weakening or restructuring the parents’ pre-existing sources of interpersonal security. The resulting experience of isolation should therefore be understood not simply as physical separation, but as a reorganization of the interpersonal field surrounding the family [4,21].

The post-treatment transition revealed another important disruption in interpersonal security. Although treatment completion is conventionally framed as a positive milestone, parents described an unexpected emotional void after leaving the hospital. The hospital had progressively become a central source of reassurance, professional monitoring, and relational support. Its sudden reduction or withdrawal could therefore generate a paradoxical sense of vulnerability: the child was clinically improving, yet parents felt less protected. From a Sullivanian perspective, this transition represents a shift in the interpersonal environment in which anxiety had previously been contained. The end of intensive treatment did not necessarily eliminate anxiety; instead, it changed its relational context. Persistent fear of recurrence and retrospective guilt concerning delayed diagnosis may consequently reflect continuing efforts to regain a sense of interpersonal security in an environment now characterized by uncertainty and reduced professional contact [37].

In cases of terminal disease progression, bereavement represented the most profound disruption of the parent’s primary interpersonal bond. The death of the child not only produced grief, but also fundamentally altered the interpersonal world in which the parent had organized meaning, identity, and emotional security. From Sullivan’s theoretical perspective, the intensity of this loss can be understood in relation to the centrality of the parent–child relationship within the individual’s interpersonal system. The disappearance of this relationship creates a profound disruption that cannot be reduced to the absence of the child as an individual; it also involves the loss of an essential interpersonal context through which the parent’s emotional life had been organized [8,38].

Within this context, the maintenance of continuing bonds, such as speaking to the deceased child, preserving memories, or maintaining the child’s legacy, can be interpreted as an attempt to reconstruct interpersonal continuity following an otherwise devastating rupture. Rather than representing an inability to adapt to bereavement, these continuing bonds may provide a relational bridge between the previous and present interpersonal worlds, allowing parents to preserve a meaningful connection with the deceased child while gradually reorganizing their sense of self and security. From a Sullivanian perspective, maintaining an internalized relationship with the deceased may therefore contribute to the reconstruction of interpersonal coherence after the loss of the primary interpersonal bond [38,39].

Taken together, these findings suggest that the parental experience of childhood cancer is not adequately understood solely in terms of individual psychological responses to illness. Sullivan’s interpersonal theory provides a framework for interpreting the entire trajectory as a dynamic process of managing anxiety and preserving interpersonal security within changing relationships [8]. Before diagnosis, parents sought validation and reassurance from healthcare professionals; during diagnosis, they attempted to contain overwhelming anxiety; during treatment, they regulated their emotional expression to protect the child and sustain the parent–child relationship; during hospitalization, their interpersonal networks were disrupted; after treatment, the reduction in professional contact generated a new form of uncertainty; and following bereavement, continuing bonds provided a means of maintaining relational continuity after the loss of the primary relationship. Thus, the findings indicate that parental adaptation to childhood cancer is fundamentally relational, involving continuous attempts to preserve, negotiate, or reconstruct interpersonal security in response to profound changes in the family’s relational environment [40].

The heterogeneity of the clinical trajectories represented in this study may warrant careful interpretation of the findings. Participants’ children differed in diagnosis, age, treatment trajectory, and disease status, and these factors inevitably shape the specific demands and stressors experienced by families. Therefore, the five axes and twelve categories should not be interpreted as diagnosis-specific or universally applicable patterns. Rather, they represent recurrent experiential meanings identified across heterogeneous individual circumstances. The heterogeneity of the clinical trajectories represented in this study may warrant careful interpretation of the findings. Participants’ children differed in diagnosis, age, treatment trajectory, and disease status, and these factors may shape the specific demands and stressors experienced by families. Therefore, the five axes and twelve categories should not be interpreted as diagnosis-specific or universally applicable patterns. Rather, they represent recurrent experiential meanings identified within the heterogeneous circumstances represented in this sample. The qualitative interview approach allowed us to explore how participants described and interpreted their experiences, while Sullivan’s interpersonal framework provided a relational lens for understanding recurring processes involving interpersonal security, emotional regulation, family relationships, and interactions with healthcare professionals. These processes should not be interpreted as evidence that parents of children with different cancers experience the illness in identical ways.

The contribution of Sullivan’s interpersonal theory to the present study extends beyond providing a theoretical background for the interpretation of findings. The theory informed the understanding of parents’ narratives by directing attention to interpersonal anxiety, the search for security, emotional regulation within significant relationships, and disruptions in the interpersonal environment. Consequently, experiences such as diagnostic denial, emotional suppression, perceived isolation, post-treatment vulnerability, and continuing bonds after bereavement were interpreted as relational processes rather than exclusively intrapsychic responses. This theoretical perspective helped integrate apparently distinct experiences across the illness trajectory into a coherent interpersonal process in which parents continuously attempted to preserve or reconstruct security within changing relationships [8].

In conclusion, while the existing literature extensively documents that childhood cancer induces anxiety, financial hardship, and fear of relapse, applying Harry Stack Sullivan’s interpersonal theory of psychiatry to Spanish families offers new theoretical perspectives that go beyond mere descriptions of parental suffering.

1. Reconceptualization of denial and emotional suppression as relational protection mechanisms. Instead of considering denial and emotional suppression as individual cognitive avoidance or maladaptive coping strategies, Sullivan’s theoretical framework reveals them as active mechanisms of interpersonal buffering. Participants’ accounts suggested that maintaining a sense of emotional stability was perceived as an important way of protecting the child during treatment. Parents intentionally compartmentalize their despair, crying only when they are alone or when the child is asleep, to project an unshakeable facade of security. Thus, emotional suppression functions as a deliberate interpersonal shield: parents absorb the relational impact to preserve the child’s psychological equilibrium, which explains why parental exhaustion peaks not during acute crises, but during the calm transitions following treatment.
2. A unique contribution of this study lies in the application of Sullivan’s interpersonal constructs to the sociocultural context of Spanish families, historically rooted in strong familism and extended intergenerational care. While Sullivan postulated that interpersonal security depends primarily on the nuclear unit, our findings show that in Spain, the extended family, particularly grandparents, is quickly integrated into the security apparatus. Grandparents assume custody of healthy siblings and manage household chores, allowing parents to isolate themselves in the hospital. However, this culturally mediated dynamic creates a double interpersonal paradox. While it provides vital structural relief, it simultaneously triggers intense parental guilt over the relational displacement of healthy siblings, disrupting overall family cohesion.
3. Previous research has defined remission anxiety primarily as an individual psychological fear of cancer recurrence. However, from Sullivan’s perspective, remission represents a systemic interpersonal detachment. During treatment, the clinical oncology team and the hospital ward become integrated into the family’s daily routine, functioning as a vital refuge from existential threat. Medical discharge abruptly severs this institutional bond, leaving parents in an unexpected relational vacuum without ongoing clinical monitoring. Therefore, post-treatment anxiety is not simply a fear of relapse, but an acute reaction to the sudden disappearance of the institutional safety net that had sustained the family throughout the illness [8,30,32,34,40].

### 4.1. Potential Implications for Clinical Practice

Although this exploratory study was not designed to evaluate clinical interventions, the participants’ accounts suggest several areas that may be relevant to pediatric oncology practice and that could inform future research.

First, the participants’ descriptions of uncertainty before diagnosis suggest that communication between families and healthcare professionals may be an important area for further attention. Greater recognition of parental concerns and clear communication regarding diagnostic processes may potentially contribute to a stronger sense of interpersonal security.

Second, the findings suggest that peer and family support may represent valuable sources of emotional and practical support. Parent-to-parent support initiatives could therefore be explored as a potential complement to professional psychosocial services, particularly during periods of prolonged treatment and hospitalization.

Third, the participants’ accounts indicate that parents may experience emotional distress that is not always directly expressed during their child’s treatment. These findings may support further investigation of proactive psychosocial assessment and opportunities for parents to discuss their emotional needs within pediatric oncology services.

Fourth, the transition from active treatment to follow-up may represent a particularly relevant period for psychosocial support. Parents’ descriptions of fear surrounding follow-up appointments suggest that survivorship programs could consider the emotional and relational dimensions of this transition.

Finally, the account of bereavement in this study suggests the potential value of continuity of psychosocial and bereavement support after the death of a child. However, because only one participant represented this experience, these observations should be regarded as hypothesis-generating rather than as evidence supporting a specific bereavement potential implication.

### 4.2. Strengths and Limitations

This study possesses several methodological strengths, including adherence to COREQ reporting standards, which includes participant review of transcripts and significantly enhances validity, rigorous investigator triangulation, and explicit integration of an established interpersonal theoretical framework. However, several limitations must be noted. First, the small sample size (*n* = 6), while methodologically justified by thematic stability in qualitative inquiry, restricts transferability to broader populations. While the sample was sufficient to achieve thematic stability within the experiences represented in this sample regarding parents’ general interpersonal experiences, some specific clinical subgroups were underrepresented. In particular, only one participant had experienced the death of a child, and only two had children in active treatment. Our findings in these areas should be interpreted as exploratory. Furthermore, the experiences of parents with children in palliative care could be included, something that is not carried out in the study’s geographic region. Second, and crucially, recruitment was conducted exclusively through a parent association. This strategy introduces a significant selection bias, as participants had already established contact with formal peer and psychosocial support networks. Consequently, the findings may underrepresent the severe isolation, unmitigated financial strain, and response deficits experienced by families with limited, reduced, or non-existent social support structures. Other associations could not be contacted because none exist in that geographic area. Third, although paternal inclusion was a strength, the higher proportion of fathers relative to mothers may influence the relational themes identified. Future research should utilize multi-center recruitment strategies across diverse clinical and socioeconomic settings, intentionally recruiting non-associated families and incorporating sibling perspectives. Fourth, because the interviews addressed potentially distressing experiences, a safety procedure was established for participants that allowed them to pause or interrupt the interview if continued participation seemed likely to cause significant emotional distress. During the seventh interview, the participant experienced considerable emotional distress related to its content. Following a discussion between the participant and the interviewer, the interview was stopped before its completion, and the resulting material was not included in the final analysis. This decision was made primarily to protect the participant from potential harm. However, this may have introduced selection bias. This possibility was considered in the interpretation of the results and is acknowledged as a limitation of the study.

## 5. Conclusions

This exploratory qualitative study suggests that parents’ experiences of childhood cancer are shaped by changes in interpersonal relationships, emotional regulation, perceived security, and support networks across different phases of the child’s illness. Participants described uncertainty and emotional disruption around diagnosis, the coexistence of perceived strength and distress during treatment, changes in family and social relationships, continuing concerns following remission, and profound grief and continuing bonds following the death of a child.

Sullivan’s interpersonal theory provided a useful conceptual lens for interpreting these experiences as relational processes involving the preservation, disruption, and reconstruction of interpersonal security. However, the small and heterogeneous sample means that these findings should be considered exploratory and should not be interpreted as exhaustive or universally applicable across the childhood cancer trajectory.

The findings also suggest potential areas for psychosocial support, including attention to parental emotional needs, peer and family support, transitions following treatment, and bereavement. These should be regarded as potential implications generated by the participants’ accounts rather than as interventions demonstrated or validated by this study. Future research involving larger and more diverse samples is needed to further examine these experiences and to evaluate specific psychosocial approaches.

## Figures and Tables

**Table 1:** Characteristics of the participants (*n* = 6).

Participant Code	Parent Gender	Parent Age (Years)	Educational Level	Occupation/ Work Status	Socioeconomic Status	Child Age (Years) & Gender	Diagnosis	Treatment Duration	Current Child Status
**EO1**	Father	41	Secondary Education	Civil servant (Requested unpaid leave)	Middle	16 years, Male	Medulloblastoma	18 months	Remission
**EA2**	Mother	38	University Degree	Employee (Took medical leave for caregiving)	Low–Middle	6 years, Female	Acute Lymphoblastic Leukemia	24 months	Remission
**EO3**	Father	43	Vocational Training	Employed (Adapted work schedule)	Middle	17 years, Female	Nongerminoma	12 months	Active Treatment
**EA4**	Mother	36	University Degree	Employed (Reduced hours)	Middle	10 years, Female	Acute Lymphoblastic Leukemia	24 months	Remission
**EO5**	Father	42	Secondary Education	Employed	Low-Middle	ᵻ 9 years Male	Embryonal Sarcoma of the Liver	8 months	Deceased
**EO6**	Father	38	Vocational Training	Self-employed (Reorganized business)	Middle	16 years, Male	Pilocytic Astrocytoma	14 months	Active Treatment

ᵻ: Deceased; EO: Male interviewee; EA: Female interviewee.

**Table 2:** Axes and categories of parents’ experience of their child’s cancer according to Sullivan’s psychological model.

Axes	Categories	Definition of Categories	Quotations
**Diagnosis**	Experiences before diagnosis	The set of experiences, emotions, and perceptions that a person goes through while dealing with symptoms and uncertainty	EO1: […she was losing her balance a little, moving about her leg in weird way.] […I suspected they were looking for some kind of tumor…] EO3: […she couldn’t see well and we thought it had something to do with her eye…] EA4: [I asked if she could have blood work in case she had some anemia. The pediatrician’s response was that children don’t have anemia and that she looked fine…] […a bone marrow transplant came to mind…]
	Experiences in diagnosis	Emotional, cognitive, and social experiences that arise when receiving and processing confirmation of the disease	EO1: […the worst news I’ve ever had in my life…] EA2: […my world fell apart…] EO3: […they tell you everything there is to know and all the options and possibilities…] EA4: [Terrible. The news of cancer is itself devastating, imagine in a child.] […it wasn’t enough. I don’t think they gave me much information…] […the hematologist couldn’t find the words to tell me. I was the one who asked her, does he have leukemia?] EO5: […they didn’t deceive us at any point. They told us everything there was to know and about the chemotherapy treatment they gave him…] […I didn’t want to believe it…] […I even told the doctor to take another look at her papers because I thought they weren’t my son’s.]
**Treatment**	Positive aspects during treatment	The positive experiences, lessons learned, or changes that can arise from disease and coping with it	EO1: […you have to be strong for him…] […we normalized it and it wasn’t just thinking, you have cancer, but that life goes on and you have to keep doing things.] […when I shaved his head, I shaved mine too. I said I was going to be with him throughout the treatment, that if he didn’t have hair, I wouldn’t have it either so he didn’t feel bad or grow a complex out of it…] EA2: [I accepted it, whatever were to happen was out of my hands…] [I assumed the role of bringing this forward…] EO3: […you have no choice but to say, well, that’s the way it is. What needs to be done and tell me how we can do it so my little girl comes out as well as possible…] […hospitalized, that’s the peace of mind you have left…] EA4: […for your child, in the end, you find strength where there is none…] EO5: […all I could think about was my child and him pulling through. That he would fight, that he would never stop fighting…] EO6: […keep living as normal as possible, because you still have other children and there’s no better way to move forward than to keep yourself busy…]
	Negative aspects during treatment	Unfavorable emotional, physical, and social consequences accompanying the disease	EO1: [You are not the father who is helping him as you have been until now, no. And that generates a feeling of helplessness, also of having to depend 100% on someone else to save your son] […the thought that hurt me the most was thinking that we could lose him or that he would be so badly affected as not to be himself any more…] […I suppressed it, I kept it locked away…] […the worst is when night comes and you’re alone…] […they finally put a nasogastric tube to feed him. He was then emaciated, his skin of a different color, with dark circles under his eyes. His skin, even though he was thin, felt very dry. I don’t know, he looked like a very sick and very thin child. He came down to 22 or 23 kg…] EA2: […I said, clench your teeth and visit the bathroom when the tears come, but don’t cry in front of her, because I didn’t want her to see that…] […I haven’t cried about it…] […I don’t know if my emotions are numb…] […it caught me completely alone…] EO3: […the helplessness of not being able to do anything. Seeing she was doing poorly, very bad and not being able to do anything.] EA4: […all I wanted was for the girl to fall asleep so I could start crying…] […in 15 days her hair fell out and of course she’s a girl, we know hair is the smallest of our worries, of course, but in the end she was a girl with long hair.] EO5: [I think what I mostly felt was anger…] EO6: […you think about the bad stuff, like, wow, this is really serious. There are a lot of people who don’t get out of this.] […we see that many of these children have the chance to have their tumor removed and go on with their lives. It makes me angry that he doesn’t have that chance…]
	Experiences in hospital admissions	Personal and emotional experiences experimented while in hospital for children to receive treatments or care related to their disease	EO1: [We let the medical team be our guide, completely…] [In my mind, I am the least important…] [His 12th birthday was spent in the hospital…] EA2: [I got behind the helm of the family boat, not only my husband, but also my mother…] […none of us went out for 47 days straight, and I said if I get through this, I will never ever get crazy, because it was terrible.] […it was like being in prison with sticks, saying, my God, how many days have I been here?] EO3: [They do everything in their hand to make you feel comfortable…] EA4: [A nurse came and maybe one day she played with her…] […we decided to have her birthday there, which was a blast…] EO6: […the hospital is a killer. Did you ever meet anyone who wants to be there?]
	Common positive experiences after completing treatment	Positive experiences and perceptions that emerge after overcoming the active phase of a child’s illness	EO1: […we gave talks at a high school…] […very proud. He was so brave throughout the treatment, very brave, because he was also aware of everything…] EA2: [I talked about it with other mothers. I said, I don’t know if this happens to you as well, but I am in a café with my work colleagues and wasn’t interested at all in their talk.] EA4: [You value more certain things that you might not have considered important before. You cherish other things…] [We’ve formed a group and we get together and do activities and, well, we never stop and continue to help other children.] EO5: […he taught us a life lesson…] […it really fulfills me…]
	Common negative experiences after completing treatment	Unfavorable experiences and emotions that may persist or appear once the active phase of treatment for the disease has ended	EO1: [I still find it hard to say. Well, my son was going to die and I couldn’t imagine what life could be without him…] EA2: […the treatment ends and you feel like you’ve been let go…] […it was a very weird void…] EA4: […how could I not regret that? I don’t know whether my daughter’s anemia could have been cured with iron supplements and we would have never ended up with leukemia.]
**Psychosocial dimension**	Family adaption	Emotional, practical, and relational adjustment process that members undergo to cope with the changes imposed by the disease	EO1: […asked to be taken off the substitutes’ list for a year at first, then I prolonged it for another eight months…] […we watched animal videos in YouTube with my child…] EA2: […my mother had to leave her life there and come here…] [I took sick leave to care for a child…] EO3: [She has a little sister who had to go live with my parents at that time and spent five months away from home…] […I did sports to escape,…it was the only time I could disconnect from the hospital and everything that was going on…] EA4: […we’ve stuck together at home…] […I had another little one, you know. You say you won’t be able to spend all your time with her.] […running was a bit of a break for me…] EO5: […we’ve supported each other not 100%, but 200%…]
	Needs and support during the process	Requirement of emotional, informational, practical, or financial assistance to cope with the impact of the disease on their life	EO1: […reduced mobility license for the car. Financial assistance available, as you had to quit your job, they give you a pension around 100 euros a month…] […we spent all our savings…] EO3: […the associations people came and said, hey, we’ll take care of the girl this afternoon.] EA4: [Sometimes you need outside help to understand many things and make your life easier.] EO5: [She helped me a lot, giving me the tools I needed to work on them…] EO6: […only someone who is really risking their loved ones’ life can understand you.] […finding us an apartment, financial assistance for gas…] [We are self-employed and we had to change the way we work so we could take care of the house, the children, everything.]
	Religious beliefs	Faith, values, and spiritual practices that guide their way of interpreting, coping with, and making sense of the disease	EA2: […I was thinking all the time, God, I trust you… I needed to believe there was something beyond what we could control] EO5: [I couldn’t go to see my Christ because I was angry with him.] EO6: [I believed in everything. Let me tell you that I prayed to all of them that he would survive.]
**Remission**	Experiences in case of remission	Emotional, cognitive, and social experiences that arise with the confirmation of improvement or cure	EO1: [When the date of follow-up appointments is approaching, let it be blood tests, MRIs, or whatever, there’s always fear.] EA4: […sometimes you see that things are going well, but you look around and see people suffering relapses…]
**Death**	Experiences when the child dies	Emotional, cognitive, and social experiences that arise in the face of permanent loss	EO5: [I want to die and be with my son…] […I want to die because my son is so young and I don’t know where he is, and I want to be with him to protect him.] […he is protecting us from up there.] […my son has left a legacy we have to keep…] […he comes to visit us from time to time. We talk to him…] [I just told the oncologists, the only thing I wanted for my son was that he wouldn’t be in pain, would not suffer, and wouldn’t know anything.]

EO: Male interviewee; EA: Female interviewee.

## Data Availability

The original findings presented in this study are included in the article. Should you have any further queries, please contact the corresponding author.
